# Fractal Analysis and Hurst Parameter for Intrapartum Fetal Heart Rate Variability Analysis: A Versatile Alternative to Frequency Bands and LF/HF Ratio

**DOI:** 10.1371/journal.pone.0136661

**Published:** 2015-08-31

**Authors:** Muriel Doret, Jiří Spilka, Václav Chudáček, Paulo Gonçalves, Patrice Abry

**Affiliations:** 1 Department of Obstetrics and Gynaecology, Hospices Civils de Lyon, Hôpital Femme-Mère-Enfant, Bron, France; 2 Physics Department, CNRS, ENS Lyon, France; 3 Czech Institute of Informatics, Robotics and Cybernetics, Czech Technical University in Prague, Prague, Czech Republic; 4 Computer Science Department, INRIA, ENS Lyon, France; CNRS, FRANCE

## Abstract

**Background:**

The fetal heart rate (FHR) is commonly monitored during labor to detect early fetal acidosis. FHR variability is traditionally investigated using Fourier transform, often with adult predefined frequency band powers and the corresponding LF/HF ratio. However, fetal conditions differ from adults and modify spectrum repartition along frequencies.

**Aims:**

This study questions the arbitrariness definition and relevance of the frequency band splitting procedure, and thus of the calculation of the underlying LF/HF ratio, as efficient tools for characterizing intrapartum FHR variability.

**Study Design:**

The last 30 minutes before delivery of the intrapartum FHR were analyzed.

**Subjects:**

Case-control study. A total of 45 singletons divided into two groups based on umbilical cord arterial pH: the Index group with pH ≤ 7.05 (*n* = 15) and Control group with pH > 7.05 (*n* = 30).

**Outcome Measures:**

Frequency band-based LF/HF ratio and Hurst parameter.

**Results:**

This study shows that the intrapartum FHR is characterized by fractal temporal dynamics and promotes the Hurst parameter as a potential marker of fetal acidosis. This parameter preserves the intuition of a power frequency balance, while avoiding the frequency band splitting procedure and thus the arbitrary choice of a frequency separating bands. The study also shows that extending the frequency range covered by the adult-based bands to higher and lower frequencies permits the Hurst parameter to achieve better performance for identifying fetal acidosis.

**Conclusions:**

The Hurst parameter provides a robust and versatile tool for quantifying FHR variability, yields better acidosis detection performance compared to the LF/HF ratio, and avoids arbitrariness in spectral band splitting and definitions.

## Introduction

Intrapartum fetal heart rate monitoring is widely used to predict fetal asphyxia, which is responsible for severe adverse neonatal outcomes [1, 2]. Monitoring fetal heart rate significantly decreases neonatal seizure and mortality, at the price of a dramatic increase in operative delivery, especially cesarean sections for suspected asphyxia, due to low specificity [3, 4]. Variability has been identified as a key element in fetal heart rate analysis [5, 6]. Classically, fetal heart rate analysis is performed visually, with high inter- or intra-observer variations [7–9].

By analogy with adults, spectral analysis has been proposed to objectively evaluate heart rate variability (HRV) and detect pathological conditions in fetuses [10–18]. In the context of HRV analysis, spectrum estimation is grounded on the use of a frequency band splitting procedure, which relies on a priori defined frequency bands. For adults, it is commonly assumed that such frequency bands are associated with autonomic nervous system activity. Notably, the low-frequency (LF) band, ranging from 0.04 Hz to 0.15 Hz is associated with sympathetic activity or sympathetic and parasympathetic activity, while the high-frequency (HF) band ranges from 0.15 to 0.4 Hz and corresponds mainly to parasympathetic activity [19, 20]. The LF/HF ratio, which consists of the ratio of the powers measured within each band, is then used to quantify the sympathovagal balance [19, 21]. This association between frequency bands and activity of both components of the autonomic nervous system is much less documented and far more debatable for fetal heart rate variability. In the fetus, higher heart rate frequency, immaturity of the autonomic nervous system, differences between maturation of the sympathetic and parasympathetic systems, an intermittent breathing cycle with high respiratory frequency, regular uterine contractions during labor increasing the intrathoracic, intra-abdominal fetal pressure and umbilical cord pressure, are among the many factors that may dramatically affect the heart rate and thus power repartition along the frequencies [22, 23]. This naturally raised the question of the relevance of the definition of these frequency bands for analyzing fetal HRV [24], which has been interestingly conducted in neonates [23].

Fractal analysis has recently emerged as method for analyzing heart rate variability beyond spectrum estimation. It has been evaluated in numerous studies for adult HRV, with sometimes controversial conclusions [25–28] but also for intrapartum fetal HRV analysis [29, 30]. Formal connections between Fourier and fractal analysis have been discussed in [31, 32], and more specific relations between fractal exponents and LF/HF-type ratio were already interestingly discussed in [25].

The aims of the present study were to investigate the relevance of the LF/HF ratio, as well as that of the intrinsically underlying frequency band-splitting procedure, which is generally driven by adult-band definitions, and to investigate the relevance of the fractal paradigm and the Hurst parameter as possible alternatives for characterizing intrapartum fetal HRV. Since hypoxia modulates the sympathetic-parasympathetic balance, which is traditionally measured with the LF/HF ratio, LF/HF ratios based on different intermediate splitting frequencies were measured and compared against Hurst parameters for a database of normoxic and hypoxic fetuses.

## Methods

Intrapartum fetal heart rate recordings were selected from a cohort collected during an ongoing study that began in 2000 in the Department of Obstetrics at the public academic hospital Femme-Mère-Enfant (HFME, Bron, France), a tertiary referral center for high-risk pregnancies, fetal medicine, and neonatology, with approximately 4500 deliveries per year [33]. An observational study was performed without any intervention. Only data regarding routine medical care were used for the study. French law requires only that patients receive written information (provided at the first medical visit) related to the ongoing research, and written consent is not needed. This procedure was approved by the institutional ethics committee of Hospices Civils de Lyon—Comité de protection des personnes (CPP) Sud-Est III. Recordings were performed during labor as part of the standard care for women with a singleton pregnancy, gestational age ≥ 37 weeks and presenting with a high risk of fetal asphyxia. High risk was defined as maternal chronic pathology, pregnancy complications, postdate delivery, fetal heart rate anomalies during labor, or meconium-stained amniotic fluid. Labor and delivery were managed according to the STAN clinical guidelines [34]. Umbilical artery pH and neonatal outcome were systematically documented. Exclusion criteria were multiple pregnancies, major fetal malformation or chromosomal anomalies, or acute event preceding birth that caused neonatal acidosis (e.g. abruption placenta, shoulder dystocia).

Cases for study were selected by the first author (M.D.) and anonymized for any further analysis. The selection was based on the neonatal acid-base status (acidosis or not) and fetal HRV classification following the blackInternational Federation of Gynecology and Obstetrics (FIGO) criteria (including baseline frequency, variability, and characteristics of decelerations if presented) [34]. Patients were eligible for the study when the fetal heart rate recording lasted longer than 30 minutes, was stopped no earlier than 30 minutes before delivery, had less than 10% of missing data, and all clinical data were documented. Cases in the Index group were fetuses with neonatal acidosis, defined as umbilical cord artery pH ≤ 7.05. This pH cutoff value was chosen since it is associated with major fetal and neonatal morbidity and mortality rates [35]. Two controls were included for each case in Index group. The 30 controls were defined as umbilical cord artery pH > 7.30 and consisted of two subgroups. One control subgroup had a normal fetal heart rate (*n* = 15), and one control subgroup had an abnormal fetal heart rate (*n* = 15).

Fetal ECGs were collected using a scalp electrode and recorded with a STAN S21 or S31 monitor at 12-bit resolution and a 500 Hz sampling rate. RR intervals were extracted from the stored STAN data by Neoventa Medical (Neoventa, Medical AB, Molndal, Sweden).

For each subject, the last 30 minutes before delivery were analyzed. Given the high quality of the selected data, basic preprocessing was needed. For each subject, missing beats were interpolated using a standard sliding median filter. RR interval list in ms, {*t*_*i*_, *i* = 1, … *I*}, were transformed into a regularly sampled time series *x*_*k*_, *k* = 1, …, *K* = *f*_*s*_ × 30 × 60 in Beats-per-Minute (BpM), with cubic spline interpolation of the series (*t*_*i*_/1000, 60000/(*t*_*i* + 1_ − *t*_*i*_)). Because the intrapartum fetal heart rate does not contain information beyond 3 Hz, the time series were resampled with the sampling frequency: *f*_*s*_ = 10 Hz. It has been checked that variations of *f*_*s*_ did not modify the results reported here.

Since heart rate variability is quantified with the LF/HF ratio, reflecting the autonomic nervous system balance, fetal HRV was analyzed in normoxic and hypoxic fetuses. Hypoxia is a *quasi-experimental* condition, known to stimulate sympathetic and parasympathetic activity [36].

As a reference, long-term variability (LTV) and short-term variability (STV) were computed, excluding decelerations [37, 38]. Decelerations were automatically detected according to the procedure implemented in [39] and previously used in [30].

The temporal dynamics of the fetal heart rate variability were analyzed using spectrum analysis, with the Welch-Periodogram estimate for the power spectral density (or Fourier spectrum) Γ_*X*_(*f*), which rely on the application of the fast Fourier transform (FFT) to BpM time series (cf. Appendix spectrum estimation). The normalized and non-normalized power, and the LF/HF ratio, denoted nLF, nHF, LH, HF, and *LF*/*HF*_0.15_ were calculated using the frequency bands recommended by Task Force for characterizing adult heart rate variability [19, 20]: The LF band ranges from 0.04 to 0.15 Hz and the HF band ranges from 0.15 to 0.4 Hz [19]. These quantities are defined in the Appendix.

First, we examined the relevance of the intermediate frequency (0.15 Hz) separating the adult LF and HF bands. A collection of different LF/HF ratios, *LF*/*HF*_*f*_, was computed by varying the intermediate frequency *f* from 0.05 to 0.35 Hz (with a 0.05 Hz step), while keeping the lower and upper limits of the LF and HF bands fixed.

Then, the fetal heart rate variability was analyzed using the fractal paradigm: This contemporary analysis used to characterize the *fractal*, or *scaling*, or *scale-free* properties of the temporal dynamics of the time series. It essentially amounts to assuming that the Fourier spectrum (or PSD) Γ_*X*_(*f*) of fetal heart rate BpM time series *X* shows a power-law decay, regarding frequencies:

$$\begin{array}{c} \Gamma_{X}\left(f\right)≃C{\left|f\right|}^{-(2H-1)}, \end{array}$$ (1)

at least across a large range of frequencies *f*, where the power law exponent is controlled by the Hurst parameter *H*. This is the case for (the increments of) the representative fractional Brownian motion.

A modern and efficient method for computing Hurst parameter relies on the use of a wavelet transform [31, 32]. The wavelet transform can be introduced by viewing it as a variation of spectrum estimation, which is naturally suited to measure fractal properties in time series (cf. e.g., [31, 32]). Wavelet coefficients *d*_*X*_(*a*, *k*) are computed as comparisons with the inner product of the BpM time series *X* against a collection of wavelets *ψ*_*a*_. The wavelets *ψ*_*a*_ are obtained from the same oscillating reference pattern, the mother-wavelet *ψ*_0_, by a dilation, or change of the scale operation, of factor *a* > 0: $\psi_{a}(t)=\psi_{0}(t/a)/\sqrt{(}a)$. The wavelet coefficients *d*_*X*_(*a*, *k*) can thus be given the meaning of scale (or frequency) content of the data around time position *k*, at scale *a*. Because *ψ*_0_ must be chosen as a band-pass filter to make the definition of the wavelet transform consistent, scale *a* and frequency *f* can be related one to another as *f* = *f*_0_/*a*, where *f*_0_ is a parameter that depends on *ψ*_0_ and the sampling frequency *f*_*s*_.

The wavelet spectrum *S*(*a*), which constitutes the wavelet counterpart of the Fourier spectrum, is defined as (cf. [31, 32]):

$$\begin{array}{c} S\left(a\right)=\frac{1}{n_{a}}\sum_{k}d_{X}^{2}\left(a,k\right), \end{array}$$ (2)

where *n*_*a*_ is the actual number of wavelet coefficients available at scale *a*. For the fractal time series, with PSD as in Eq (1), blackfor mother wavelets *ψ*_0_ with satisfactory localization in the frequency domain, the wavelet spectrum *S*(*a*), behaves as a power law regarding the analysis scale *a* with a power law exponent proportional to the Hurst parameter:

$$\begin{array}{c} S\left(a\right)≃C_{\psi_{0},H}\;\;\;a^{2H-1}, \end{array}$$ (3)

across a wide range of scales *a*. Parameter *H* can be estimated by performing a linear regression in a log *S*(*a*) versus log *a* diagram, often referred to as the logscale diagram (LD), across scales *a*_1_ to *a*_2_, chosen as relevant by practitioners. The wavelet spectrum provides a more robust and more reliable estimate of parameter *H* than the classical Fourier spectrum, or PSD (cf. [31, 32]).

**Fig 1:**
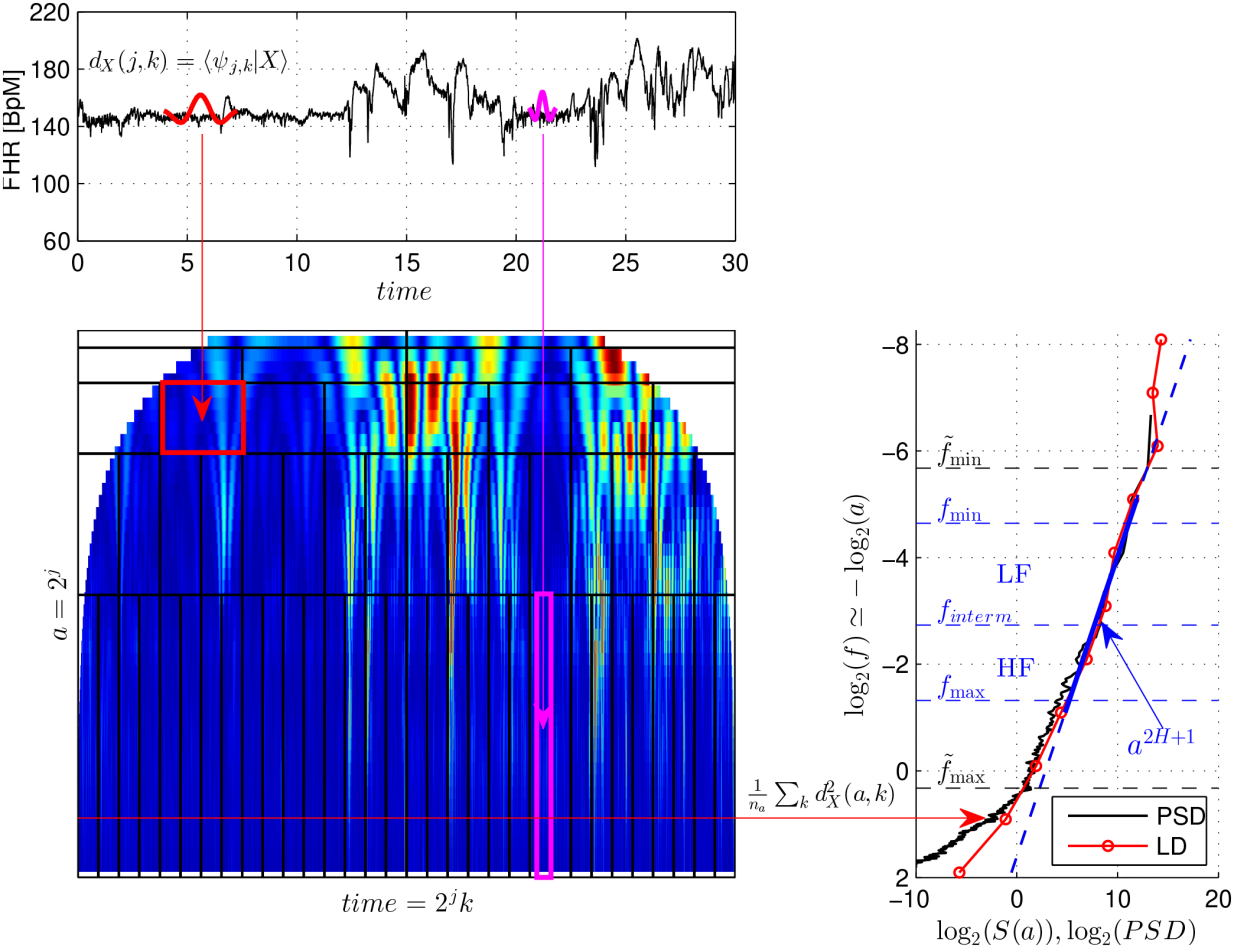
Wavelet coefficients, Wavelet Spectrum and Hurst Parameter.

The principle of the computations of the wavelet coefficients and the wavelet spectrum, together with the estimation of the Hurst parameter, is illustrated in Fig 1.

The wavelet framework for fractal and multifractal characterization has been used in the context of the intrapartum fetal heart rate variability analysis in [29, 30].

Furthermore, it has theoretically been shown that estimated Fourier ${\hat{\Gamma }}_{X}(f)$ and the wavelet *S*(*a*) could be superimposed roughly as

$$\begin{array}{c} S\left(a=f_{0}/f\right)≃{\hat{\Gamma }}_{X}\left(f\right), \end{array}$$ (4)

where *f*_0_ is a constant that depends on *ψ*_0_ (cf. [31, 32]). Furthermore, assuming an exact power law shaped PSD (as in Eq (1)) for *f*_min_ ≤ *f* ≤ *f*_max_, we also show here that the Hurst parameter *H* and the LF/HF ratio, computed using the bands LF with *f* ∈ [*f*_min_, *f*_*interm*_] and HF with *f* ∈ [*f*_*interm*_, *f*_max_], are theoretically related:

$$\begin{array}{c} LF/HF_{f_{\min},f_{interm},f_{\max}}=\frac{f_{interm}^{2-2H}-f_{\min}^{2-2H}}{f_{\max}^{2-2H}-f_{interm}^{2-2H}}. \end{array}$$ (5)

Eqs (4) and (5) are shown in detail in the Appendix (Wavelet versus Fourier Spectra). For simplicity, LF/HF_*f*_min_, *f*_*interm*_, *f*_max__ is denoted LF/HF_*f*_*interm*__ when the adult upper and lower bounds are used, i.e., *f*_min_ = 0.04 Hz and *f*_max_ = 0.40 Hz.

The acidosis detection performance was evaluated by computing receiver operational characteristic (ROC) curves for the LF/HF ratio, for modified LF/HF ratio and Hurst parameters. Statistical analysis was performed with IBM SPSS statistics 19 (SPSS Inc., Chicago, IL). Continuous quantitative data are expressed as mean and standard deviation (s.d.), and qualitative data are expressed as percentage and 95% confidence interval. Quantitative data are compared with a Student t-test and qualitative data were compared with a chi-square or a Fisher exact test as appropriate. A *p*-value < 0.05 is considered significant.

Spectral and wavelet analysis, as well as LF/HF ratio and Hurst parameter estimation, was conducted using MATLAB routines all written and designed by the authors and are available upon request.

## Results

### Subjects and fetal heart rate characteristics

Clinical data for the 45 fetuses included in the present study are reported in Table 1. As expected, the fetuses in the Index group had a lower umbilical cord arterial pH (*p* < 0.05) and underwent operative delivery for suspected fetal asphyxia more often (*p* < 0.05). Maternal data and obstetrical history for the two subgroups within the Control group were not significantly different (not shown). Birthweight was similar in the Index and Control groups thus the spectral estimation was not influenced [40]. Cardiotocograms (CTG) in the last 30 minutes were abnormal in all Index group fetuses. Basal frequency was slightly higher in the Index group whereas LTV and STV were similar for both groups (Table 1).

**Table 1:** **Clinical data and fetal heart rate characteristics** of the fetuses in the Index and the Control groups. Values are expressed as mean (s.d.) or number (%).

	Index group	Control group	*p*
	n = 15	n = 30	
Gestational age (days)	280 (8.2)	278.3 (10.3)	ns
Birthweight (g)	3312 (547)	3282 (561)	ns
Operative delivery for fetal distress (n;%)	12 (80%)	1 (3.3%)	< 0.05
Umbilical cord arterial pH	7.00 (0.03)	7.33 (0.031)	< 0.05
Umbilical cord arterial Base deficit	9.2 (2.3)	3.6 (1.9)	< 0.05
Apgar score 5 minutes > 7	100%	100%	ns
Time lag from end of recording and birth (min.)	5.40 (4.85)	7.7 (9.33)	ns
basal heart rate (bpm)	156.07 (9.35)	146.57 (15.39)	< 0.05
LTV (bpm)	19.53 (4.27)	19.20 (6.92)	(ns)
STV (ms)	6.31 (2.09)	7.65 (4.82)	(ns)

### Spectral analysis, using adult frequency bands

Table 2 reports the absolute and normalized powers for the LF and HF bands and the LF/HF ratio. The LF/HF ratio was significantly higher for the Index group compared to the Control group. Within the Control group, LF/HF ratio was not significantly different between the two subgroups in terms of normal and abnormal fetal heart rate (respectively: 4.03 (2.74) versus 4.09 (1.87); *p* = 0.43).

**Table 2:** **Spectral analysis indices** for the Index and Control groups, using adult frequency band splitting.

	Index group	Control group	p-value
LF	80.13 (49.07)	55.47 (52.83)	< 0.05
HF	10.37 (6.33)	17.51 (19.19)	ns
Total power	90.50 (51.88)	72.98 (69.39)	ns
nLF	0.87 (0.07)	0.77 (0.09)	< 0.05
nHF	0.13 (0.07)	0.23 (0.09)	< 0.05
LF/HF	8.56 (5.28)	4.06 (2.31)	< 0.05

### LF/HF ratio with different intermediate frequencies

Table 3 reports the LF/HF ratios computed by varying the intermediate frequency that separates the LF and HF bands. Whatever the intermediate frequency, the LF/HF ratio was always significantly higher for the Index group compared with the Control group. Within the Control group, the LF/HF ratios were not significantly different for the subgroups with normal and abnormal CTGs (cf. Table 4).

**Table 3:** **LF/HF_*f*_*interm*__ ratios** computed using frequency bands, LF ∈ [0.04, *f*_*interm*_] and HF ∈ [*f*_*interm*_, 0.40], with different *f*_*interm*_, in the Index and Control groups.

*f*_*interm*_	Index group	Control group	p-value
0.10	3.58 (2.04)	1.77 (1.14)	< 0.05
**0.15**	**8.56 (5.28)**	**4.06 (2.31)**	**< 0.05**
0.20	17.69 (12.71)	7.97 (5.02)	< 0.05
0.25	33.04 (24.06)	15.30 (10.01)	< 0.05
0.30	66.36 (49.80)	31.37 (19.50)	< 0.05
0.35	182.09 (140.13)	86.79 (60.43)	< 0.05

**Table 4:** **Discriminative power of LF/HF_*f*_*interm*__ ratios** computed using frequency bands, LF ∈ [0.04, *f*_*interm*_] and HF ∈ [*f*_*interm*_, 0.40], with different *f*_*interm*_, within the Control group.

*f*_*interm*_	Control group		p-value
	abnormal CTG	normal CTG	
0.10	1.85 (1.02)	1.69 (1.27)	ns
0.15	4.09 (1.87)	4.03 (2.74)	ns
0.20	7.67 (3.34)	8.27 (6.39)	ns
0.25	14.57 (5.88)	16.04 (13.12)	ns
0.30	29.18 (12.66)	33.57 (24.84)	ns
0.35	78.78 (39.79)	94.79 (76.44)	ns

**Fig 2:**
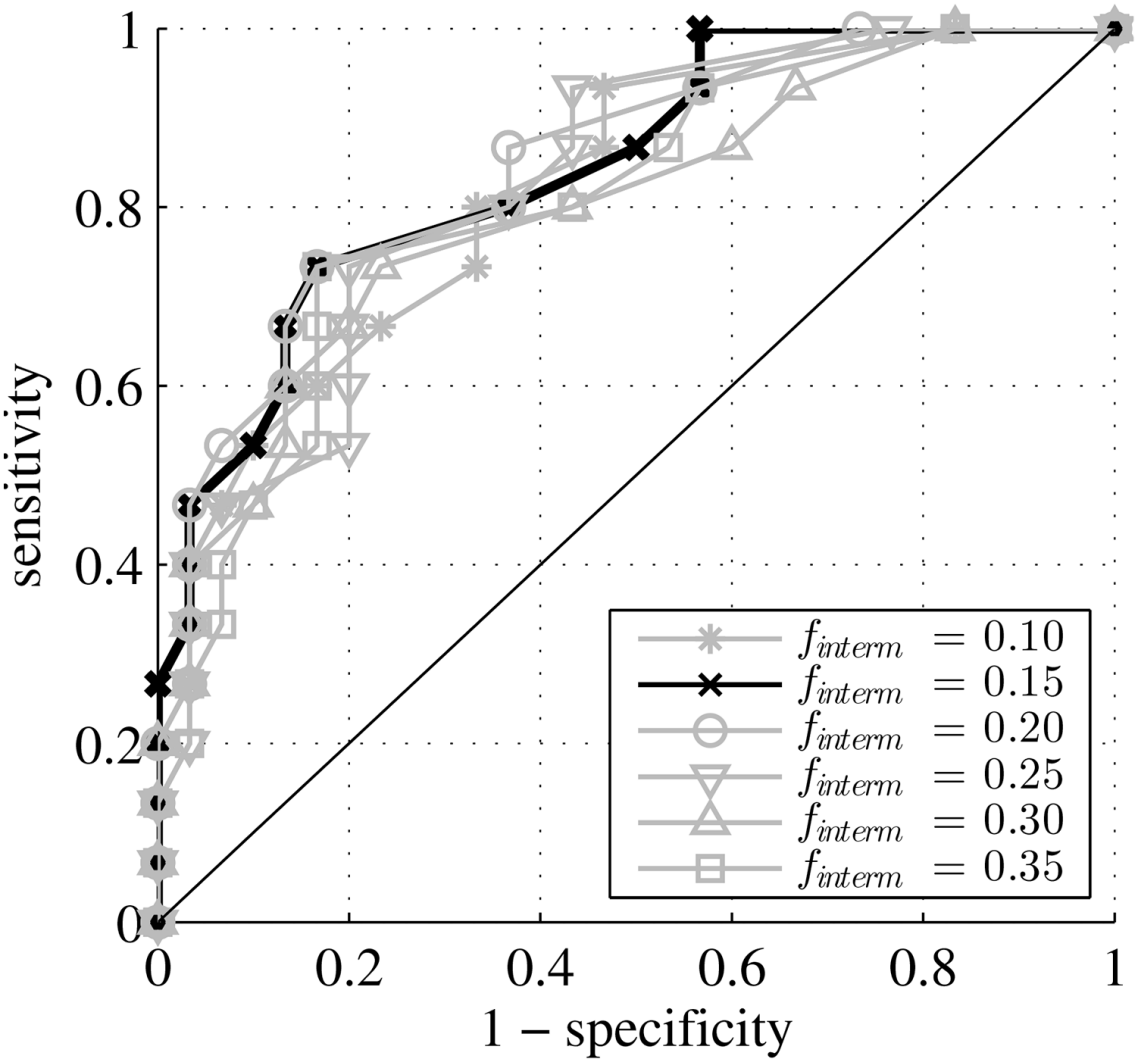
ROC curves for LF/HF ratios. ROC curve for representative LF/HF_*f*_*interm*__ ratios computed from ranges LF = [0.04, *f*_*interm*_], HF = [*f*_*interm*_, 0.40] with different intermediate frequencies *f*_*interm*_. The classical adult bands, *f*_*interm*_ = 0.15Hz corresponds to the dark black solid line.

The ROC curves reporting sensitivity and specificity of the LF/HF ratio with different intermediate frequencies are plotted in Fig 2. AUC values, shown in Table 5, were all significantly different from random classification (*p* < 0.05). In addition, the AUCs did not significantly differ from eacho ther with *p* > 0.05. Tables 2 and 5 and Fig 2 show that the LF/HF ratios computed with different intermediate frequencies are all essentially equivalent in terms of the acidosis detection performance.

**Table 5:** **Area under the ROC curve (AUC)** LF/HF_*f*_*interm*__ ratios, computed using frequency bands, LF ∈ [0.04, *f*_*interm*_] and HF ∈ [*f*_*interm*_, 0.40], with different *f*_*interm*_, in the Index and Control groups. Data are expressed as mean (s.d.), p-values quantify the statistical significance against the random classifier (diagonal on the ROC curve).

*f*_*interm*_	AUC	p-value
0.10	0.79 (0.08)	< 0.05
**0.15**	**0.82 (0.07)**	**< 0.05**
0.20	0.82 (0.07)	< 0.05
0.25	0.80 (0.07)	< 0.05
0.30	0.77 (0.08)	< 0.05
0.35	0.78 (0.08)	< 0.05

### Hurst parameter computed over the LF-HF adult frequency domain

**Fig 3:**
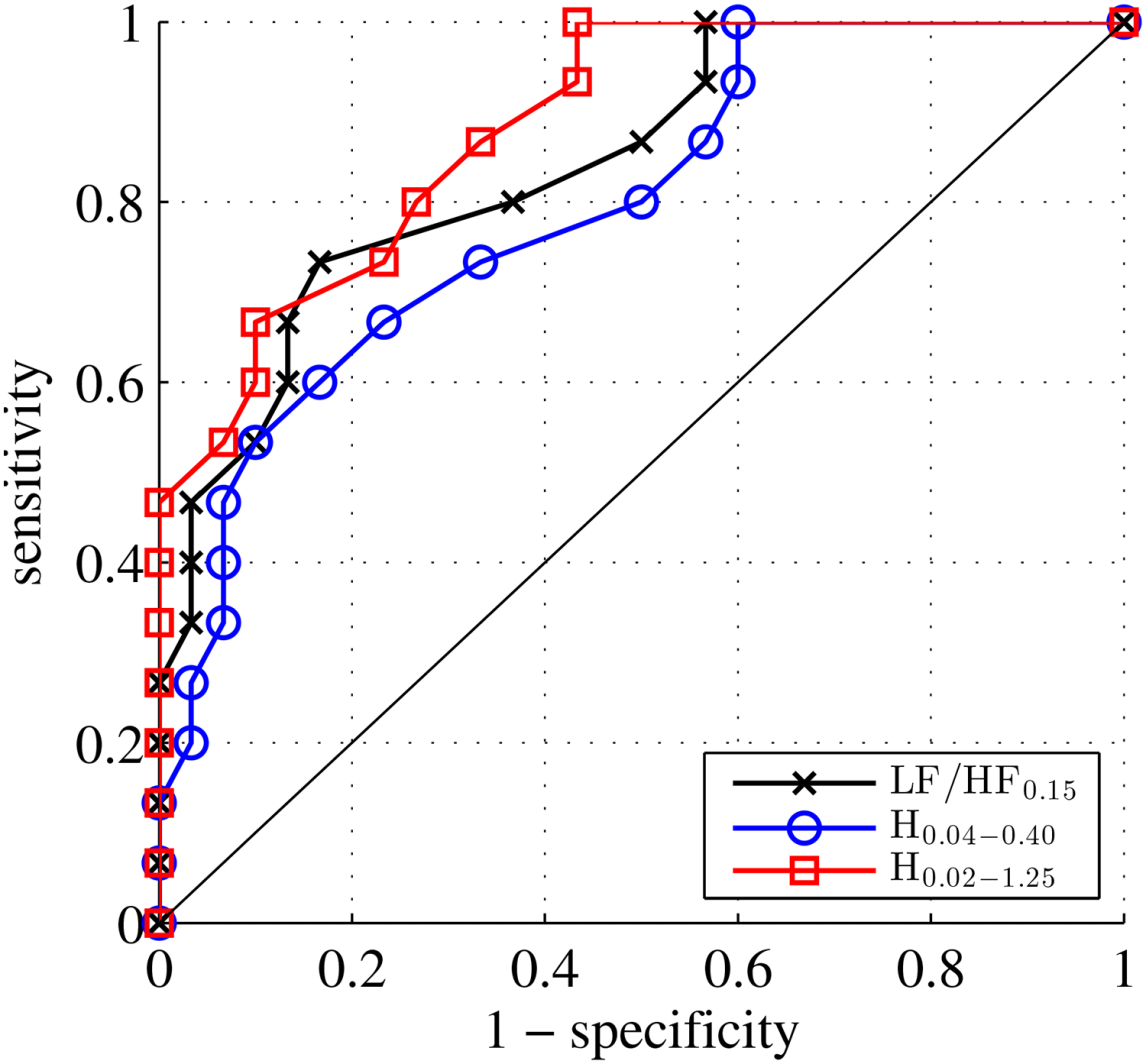
ROC curves. for ${\hat{H}}_{0.04-0.40}$ superimposed to that obtained from LF/HF_0.15_ are found to yield equivalent performance while the former avoids the recourse to the arbitrary and irrelevant choice of the intermediate frequency *f*_*interm*_ = 0.15Hz.

Parameter *H*, estimated according to the procedure described in Section 2, at scales corresponding to the frequency range 0.04 to 0.4 Hz (referred to as ${\hat{H}}_{0.04-0.40}$) were significantly higher in the Index group (0.70 ± 0.14) compared to the Control group (0.55 ± 0.13; *p* = 0.003). ${\hat{H}}_{0.04-0.40}$ did not significantly differ between the subgroups with normal and abnormal fetal heart rate in the Control group (0.56 ± 0.15 versus 0.54 ± 0.12 Hz, respectively; *p* = 0.71). For the acidosis detection performance, the AUC of the ROC curve (0.78 ± 0.08) differed significantly from the random classifier. The ROC curve obtained from ${\hat{H}}_{0.04-0.40}$ was found as significant as the one stemming from LF/HF_0.15_ (cf. Fig 3), while avoiding the selection of any intermediate frequency, thus confirming the absence of relevance of the choice *f*_*interm*_ = 0.15Hz.

### Wavelet versus Fourier spectra: Fractal dynamics

**Fig 4:**
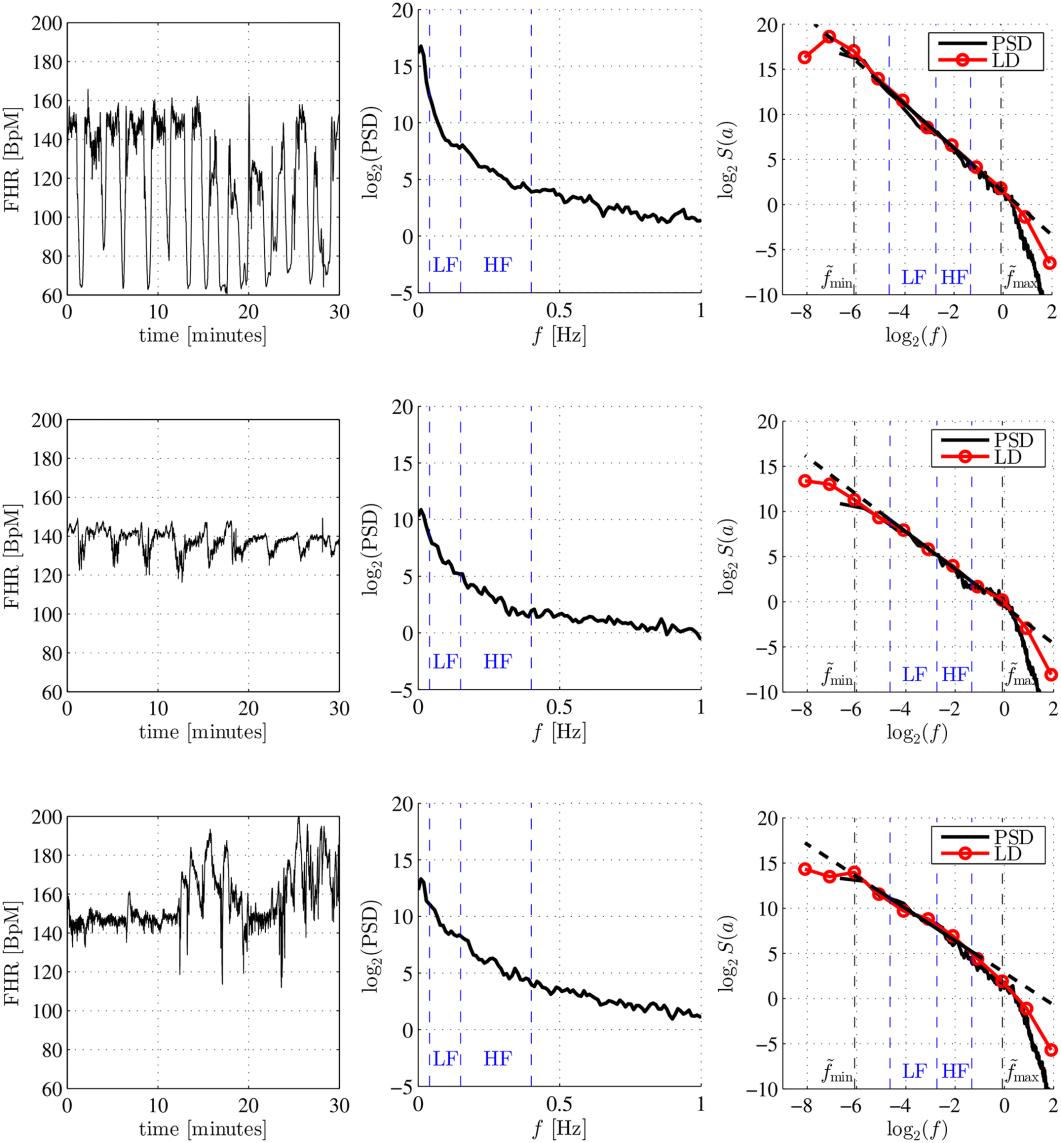
Fetal heart rate BpM time series PSD. From left to right, BpM time series, Fourier spectrum (or Power Spectral Density) and Wavelet Spectrum (Logscale Diagram (LD)), From top to bottom, example from the Index group, example from the Control group with abnormal fetal heart rate, example from the Control group with normal fetal heart rate. The power-law shaped Fourier and wavelet spectra indicate fractal dynamics, rather than frequency band-type dynamics.

For each subject, the wavelet spectrum *S*(*a*) was superimposed on the estimated Fourier spectrum (or PSD) cf. Fig 4, and they were observed to remarkably superimpose one on the other, as theoretically expected. In addition, the Fourier and wavelet spectra had similar shapes, for fetuses with and without acidosis. More precisely, Fourier and wavelet spectra had power law shape decays, in clear contrast to a two-bump curve, which had been expected to drive a frequency-band split analysis.

**Fig 5:**
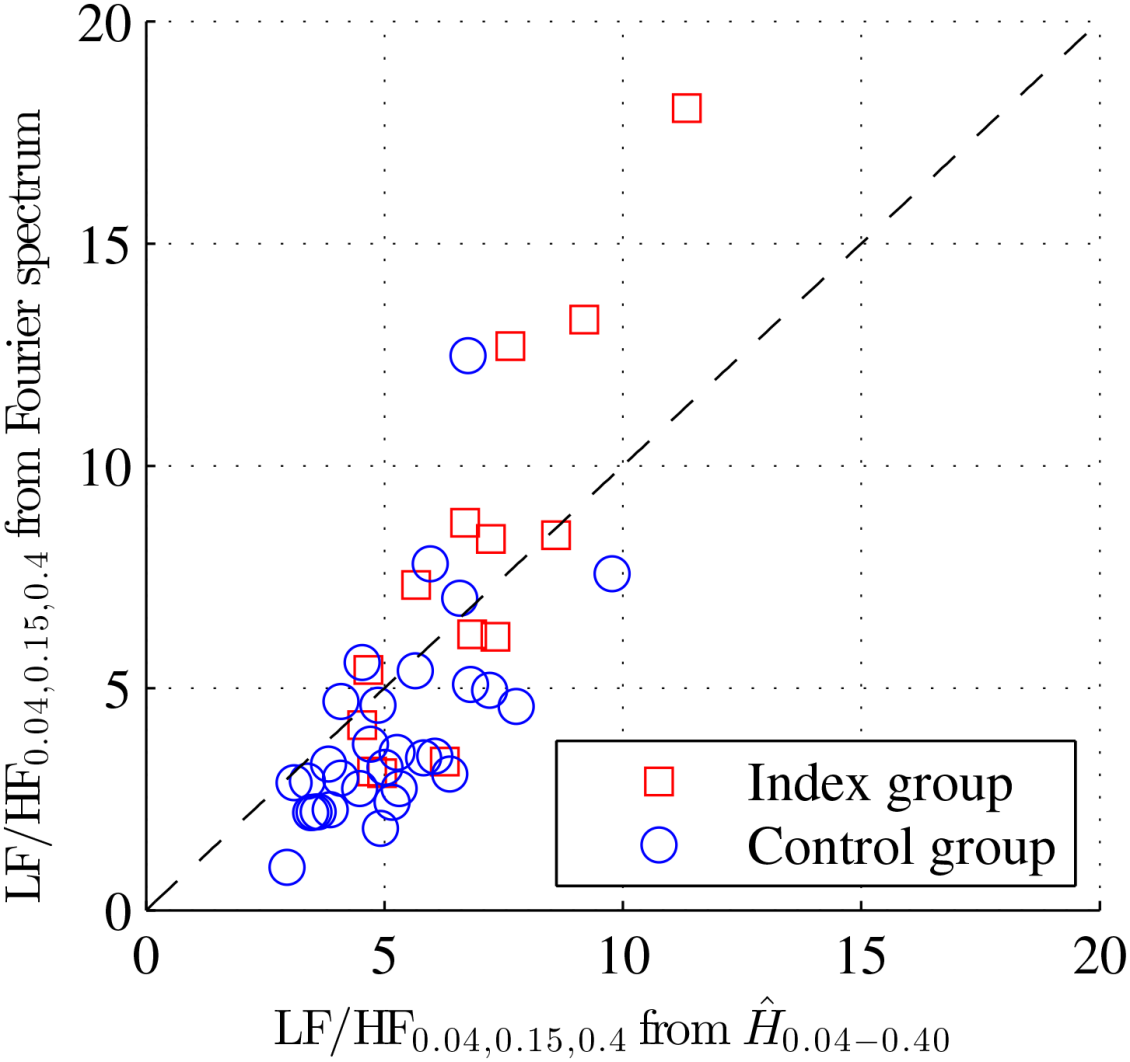
Wavelet and Fourier LF/HF ratios. The LF/HF ratio measured classically using spectral analysis is compared against the LF/HF ratio computed a posteriori using Eq (5) with the Hurst exponent ${\hat{H}}_{0.04-0.40}$, estimated via the wavelet spectrum. The very satisfactory match between both LH/HF ratios is a strong evidence indicating a power-law shaped decay of the PSD of intrapartum fetal HRV time series.

In addition, in Fig 5, the LF/HF_0.15_, measured with spectral analysis and the band splitting procedure, is plotted against LF/HF_0.15_ that was computed a posteriori by plugging ${\hat{H}}_{0.04-0.4}$ into Eq (5) above: For both groups, the match was satisfactory (with correlation coefficients of 0.69 for the Control group and 0.85 for the Index group). This is clear and strong empirical evidence supporting the claim that intrapartum fetal BpM PSD was satisfactorily modeled by a power-law behavior, as in Eq (1) above, at least within the following range of frequencies: *f*_min_ = 0.04 ≤ *f* ≤ *f*_max_ = 0.4.

### Fractal (or scaling) frequency range

A closer examination of Fig 4 also shows that the Fourier and wavelet spectra display power law behavior over a range of frequencies that extend above the upper limit and below the lower limit, corresponding to the frequency bands defined for adults. More precisely, applying a bootstrap-based goodness-of-fit test statistical test, recently devised in [41], to each subject, showed that the medians of the lower and upper bounds of the frequency range where the power-law decrease held corresponded to 0.02 Hz (thus much below the adult lower limit of 0.04 Hz) and 1.25 Hz (thus much above the adult upper limit of 0.40 Hz).

### Acidosis detection performance based on the Hurst parameter

This naturally leads to estimating the Hurst parameter on the extended range of scales corresponding to frequencies ranging from 0.02 to 1.25 Hz. The estimate denoted, ${\hat{H}}_{0.02-1.25}$, was significantly higher in the Index group (0.69 ± 0.12) compared to the Control group (0.51 ± 0.10; *p* = 0.0007).${\hat{H}}_{0.02-1.25}$ did not significantly differ between the subgroups with normal and abnormal fetal heart rate within the Control group (respectively, 0.52 ± 0.10 versus 0.51 ± 0.10; *p* = 0.74). The ROC curves obtained from ${\hat{H}}_{0.02-1.25}$ and ${\hat{H}}_{0.04-0.40}$ are compared in Fig 3, the ROC curve computed from ${\hat{H}}_{0.02-1.25}$ (with AUC = 0.87 ± 0.06) is systematically higher than from ${\hat{H}}_{0.04-0.40}$ (AUC = 0.78 ± 0.08). This difference was not significant but clearly indicates a tendency to increased power in detecting acidosis when the extended frequency domain is used.

## Discussion

The results show that the core principle of splitting frequencies into bands, inherent in the definition of an LF/HF ratio, and generally driven by adult-band definitions, is not relevant for analyzing and describing intrapartum fetal HRV temporal dynamics. First, we showed that choosing any arbitrary intermediate frequency *f*_*interm*_ for calculating the LF/HF ratios yields similar acidosis detection performance. Second, we showed that the Fourier and wavelet spectra displayed a power-law decrease along the frequencies. These results show that the principle of frequency band PSD splitting itself is not appropriate. The LF/HF ratio calculation relies on the central idea that at least two distinct spectral modes concentrating powers, corresponding to sympathovagal activity, are identifiable in the PSD. However, blockage of the sympathetic or parasympathetic nervous system in fetal sheep led to decreased spectral energy at a range of frequencies and not only in the specific frequency bands [24]. Thus, the assumption that two distinct frequency regions exist with a minimum between them guiding the choice of the intermediate frequency separating the LF and HF domains is, at least, controversial. This a priori two-bump shape was never observed in the power density spectrum computed from the intrapartum fetal BpM time series in term fetuses, whatever the acid-base status. Instead, PSDs display a power-law decay regarding frequency, which prevents a preferred intermediate frequency from being identified to define a LF/HF ratio. Similarly, power law-shaped spectra in fetuses at term before and during labor were previously reported several times [10, 11, 22, 42–44]. In contrast in spectral analysis of adult heart rate variability, two main energy concentration lobs were sometimes reported, and have specifically been correlated to autonomic nervous system activity and the baroreflex function [19, 20, 45, 46]. In fetuses, the basal frequency varies under normal conditions from 110 to 160 bpm, thus implying that fetal HRV temporal dynamics have energy in frequencies up to 1.4 Hz, in contrast to the adult basal frequency that concentrates within 50 to 80 bpm, and thus HRV dynamics up to 0.6 Hz. Therefore, as previously reported for newborns, frequency bands as defined by the Task Force for adults are not appropriate for fetuses with high basal heart rate [23].

Furthermore, compared to that of adults, the autonomic nervous system is globally immature in fetuses. A delay exists in the parasympathetic component maturation compared with the sympathetic component [47]. This could modify the PSD shape, which may be largely influenced by sympathetic or parasympathetic activity. Corroborating this hypothesis, researchers demonstrated that PSDs were different in term and preterm fetuses exposed to hypoxia [17]. Moreover, during labor, regular uterine contractions lead to high pressure variations on the fetal thorax and then the cerebral and cardiac blood pressure changes with very low frequency around 0.01 to 0.003 Hz). Additionally, fetuses have episodic, irregular, and high rate breathing movements, which influence intrathoracic blood pressure and autonomic nervous system activity [48]. In asphyxiated fetuses, breathing movement drastically decreased [49]. Therefore, many competing mechanisms and causes contribute to the regulation of intrapartum fetal HRV, due to the fetus’s ongoing development, its specific intrauterine environment, and interaction with the mother [17, 23]. These mechanisms may constitute potential explanations for the specific power-law shaped PSD and thus for the fractal dynamics of intrapartum HRV of the fetus. Fractal dynamics imply that there exist no preferred time scales that play a specific role in the temporal dynamics of intrapartum fetal HRV, or equivalently that all scales equally contribute to the temporal dynamics. The richness of fractal dynamics is thus not in what happens at specific scales, but instead in the mechanism that relates all scales together and that is quantified with the Hurst parameter *H*.

Having observed that the LF/HF ratio is poorly relevant in fetuses as well as power-law-shaped Fourier and wavelet spectra decay, we demonstrated that the Hurst parameter is a relevant alternative that renews and enriches intrapartum fetal HRV analysis.

Fourier and wavelet spectra can be theoretically related (see Eq (4) and Appendix or [31, 32]). Thus, for an exact power law PSD, the standard LF/HF ratio can be analytically related to Hurst parameter *H*, as recalled in Section 2 and Eq (5) above. In this study wavelet and Fourier spectra matched well (cf. Fig 4), as well as in the relation between the LH/HF ratio and the Hurst parameter, a non-trivial observation with real-world data. Connections between fractal exponents and the LF/HF type-ratio were made in [25], in the context of HRV analysis, relying on fractal indices computed from detrended fluctuation analysis (DFA) [50]. The present contribution thus elaborates and extends the interesting analysis in [25], by first applying it in the context of fetal intrapartum HRV analysis, and second by using a wavelet framework for estimating the Hurst parameter, instead of DFA. The use of the wavelet framework yields the following benefits. At the theoretical level, *H* is theoretically equivalent to the LF/HF ratio for power-law-shaped PSD, but its measurements does not rely on the a priori and not well-grounded choice of an intermediate frequency. At the practical level, the estimation of *H* is more robust to additive trends (such as baseline slow drifts) than Fourier-based calculation of the LF/HF ratio [32]. In addition to the benefits of using wavelets rather than DFA for robust estimation of fractal parameters, the wavelet framework permits a more solid and natural theoretical grounding of the relation between Fourier and wavelet spectra, thus relating clearly and simply fractal analysis to Fourier analysis. These comparisons have been fully detailed in previous publications [31, 32]. Using wavelet analysis also paves the way to extension towards more advanced fractal schemes, such as multifractal analysis, which extend the analysis beyond PSD, commonly used for adult HRV [26] and intrapartum fetal HRV [29].

In addition to investigating the relevance of the intermediate frequency and of the band-splitting procedure, this study also examines fractal temporal dynamics in intrapartum fetal BpM time series involves a range of frequencies larger than that prescribed by adult LF-HF bands (0.04 to 0.40 Hz). There was no physiological or empirical data-driven reasons why Hurst parameter estimation should be restricted to the use of an adult-based prescription for extreme frequencies. Instead, the wavelet framework used in the present study naturally permits a data-driven selection of the actual range of frequencies over which power law behaviors (thus fractal dynamics) hold. This enabled us to show that the frequency domain with fractal dynamics extends from 0.02 Hz to 1.25 Hz. The question of the frequency domain limit was indirectly addressed in previous studies, as some authors used different frequency domain definition to examine fetal heart rate variability [15, 17, 43, 51]. This is consistent notably with [52] and [22], who showed that significant power beyond the adult bands contribute to fetal heart rate dynamics. These additional lower and upper frequency ranges can convey information, potentially relevant for fetal heart rate characterization and acidosis detection. Interestingly, the extension of the classical LF/HF frequency range beyond 0.40 Hz toward 1 Hz is consistent with the very recent results in [53] reporting that the modulation of the power of frequencies around 1 Hz help discriminate between healthy fetuses and fetuses suffering from acidosis. In addition, these very high frequencies have been related to fetal respiratory movements [52]. To the opposite, the extension of the classical LF/HF frequency range below 0.04 Hz down to 0.02 Hz characterization, a range of frequencies related to decelerations. The importance of very low frequencies for fetal heart rate dynamics in general and for acidosis detection in particular has already been mentioned in [13, 38, 52]. These connections have, very recently, been further documented and precisely quantified in terms of relations between very low frequency and decelerations [30].

Moreover, the lower and upper bounds of the range of frequencies where the PSD power law behavior holds, ${\tilde{f}}_{\text{min}}≃0.02\leq f\leq {\tilde{f}}_{\text{max}}≃1.25$, interestingly match the time scales *a*_max_ ≃ 50s and *a*_min_ ≃ 1s, that are used to computate LTV (1 min = 60s) and STV (3.75s). Fractal analysis thus also renews the temporal-based LTV or STV measures of heart rate variability, in several respects: LTV or STV measures variability at a priori defined time scales (1 minute and 3.75 s) and decide that variability is good when the LTV or the STV exceeds an a priori selected threshold. Instead, fractal analysis involves a continuous large range of time scales with in data analysis to range from *a*_min_ ≃ 1s to *a*_max_ ≃ 50s. Variability is no longer assessed by values (of LTV or STV) measured at predefined scales that exceed a prescribed threshold, but instead by a low value of the power-law exponent, the Hurst parameter, which quantifies the relation among all scales simultaneously and continuously from *a*_min_ to *a*_max_. Fractal analysis thus can unify and extend more traditional measures of heart rate variabilities performed in the temporal and spectral domains. This has been further quantified and documented in [29, 30].

The power-law shape of the PSD indicates that the larger the *H*, the larger the contribution of low frequencies to the temporal dynamics of intrapartum fetal BpM time series. Therefore, in essence, Hurst parameter *H* acts as a frequency balance quantifying the richness of the frequency content of the temporal dynamics. The Hurst parameter can thus be interpreted as a (fractal) LF/HF balance, in the spirit of the classical LF/HF ratio, with yet two major differences: First, the upper and lower bounds of the frequency range are not defined a priori, but their selection is data driven. Second, there is no need for the arbitrary definition of an intermediate frequency separating the LF and HF components.

In the present study, we found that the Hurst parameter was significantly higher for the Index group compared to the Control group. A larger *H* thus indicates poorer frequency content in the temporal dynamics of the BpM time series in the Index group (acidotic fetuses) than in those of healthy fetuses. The Hurst parameter can thus be regarded as an index quantifying HRV: When *H* increases, HRV variability decreases, and conversely, a low *H* is a sign of large variability and thus of good health. The Hurst parameter can thus be used as an index permitting to detect intrapartum fetal acidosis [29, 30].

## Conclusions

Our study showed that choosing any arbitrary intermediate frequency *f*_*interm*_ for calculating the LF/HF ratios yield a similar acidosis detection performance. This is consistent with our observation that PSD of intrapartum fetal heart rate BpM time series do not exhibit a two-bump function, essential to support the frequency band-splitting procedure.

This study has shown that fetal heart rate BpM time series display fractal, or scaling, temporal dynamics, involving power-law decrease for frequencies ranging from very low (≃ 0.02 Hz) to very high (≃ 1.25 Hz). Fractal dynamics clearly questions the relevance of the frequency band split procedure, underlying the definition and calculation of the LF/HF ratio. Therefore, our results did not corroborate the use of the LF/HF for fetal heart rate analysis. Instead, the Hurst parameter constitutes an interesting alternative to the LF/HF ratio, avoiding the a priori and arbitrary selection of an intermediate frequency, while preserving the intuition of a power frequency balance that may be related to autonomic nervous system activity. Moreover, this study clearly showed that information relevant to intrapartum fetal HRV exists in an enlarged frequencies range, compared with the adult-based LF and HF frequency bands. Extending that classical band toward higher and lower frequencies permits better discrimination between normoxic and acidotic fetuses, improving classification performance.

This also offers the possibility that, beyond the value of *H* itself, the range of frequencies across which fractal properties holds, can in itself be a feature for detecting acidosis. Though applied to regularly resampled BpM time series, the present study performed on RR intervals times series (as is done in, e.g., [25]) is expected to yield conclusions similar in all respects. These two issues are under investigation. Further, the potential use of Hurst parameter as a relevant feature that can be involved in detecting acidosis will be investigated on a much larger database. Detection performance will be compared to those obtained from other candidate features.

## Appendix

### Spectrum estimation

It has been chosen here to use the non-parametric Welch periodogram-based estimation of the power spectral density (PSD) or spectrum Γ_*X*_(*f*) applied to

$$\begin{array}{c} {\hat{\Gamma }}_{X}\left(f=j\cdot \Delta_{f}\right)=\frac{1}{P}\sum_{p=1}^{P}{\left|\sum_{k=0}^{n_{f}-1}w\left(k\right)x\left(k+p\tau \right)e^{ı2\pi jk/n_{f}}\right|}^{2},\;\text{for}\;j=0,1,\ldots ,n_{f}. \end{array}$$

For Welch-Periodogram parameters, a Gaussian-like windowing function *w* is used with window size *n*_*f*_ = 1024 and 80% overlap (thus time shift *τ* corresponds to 20s and *P* = 83), thus yielding an approximate frequency resolution of Δ_*f*_ = *f*_*s*_/*n*_*f*_ ≃ 0.01Hz.

Following [19, 20] frequency bands for characterizing adult heart rate variability are defined as follows: The low-frequency (LF) band ranges within *f* ∈ [*f*_min_, *f*_*interm*_], while the high-frequency (HF) band ranges within *f* ∈ [*f*_*interm*_, *f*_max_], with *f*_min_ = 0.04Hz, *f*_*interm*_ = 0.15Hz and *f*_max_ = 0.40Hz.

The absolute and relative powers within each band and the LF/HF ratio are measured from the estimate ${\hat{\Gamma }}_{X}(f)$ as:

$$LF\;=\;\sum_{f_{\text{min}}\leq j\cdot \Delta_{f}\leq f_{interm}}\hat{\Gamma }\left(f=j\cdot \Delta_{f}\right),$$ (6)

$$HF\;=\;\sum_{f_{interm}\leq j\cdot \Delta_{f}\leq f_{\text{max}}}\hat{\Gamma }\left(f=j\cdot \Delta_{f}\right),$$ (7)

nLF=LF/(LF+HF), (8)

nHF=HF/(LF+HF), (9)

$$LF/HF_{f_{\text{min}},f_{interm},f_{\text{max}}}\;=\;LF/HF.$$ (10)

### Wavelet versus Fourier Spectra

Let *X* denote a second-order random process. It has been shown [31, 32] that the power of the wavelet coefficients can be related to the data PSD as

$$\begin{array}{c} Ed_{X}{\left(a,t\right)}^{2}=\int \Gamma_{X}\left(f\right)a{|\Psi_{0}\left(af\right)|}^{2}df, \end{array}$$ (11)

where Ψ_0_ stands for the Fourier transform of *ψ*_0_ and 𝔼 denotes the mathematical expectation. Therefore, the time average *S*(*a*) can be interpreted as an estimator for the ensemble average quantity 𝔼*d*_*X*_(*a*, *k*)^2^ and thus as a wavelet-based estimate of the PSD Γ_*X*_, around frequency *f* = *f*_0_/*a*, and is thus often referred to as the *wavelet spectrum*, *f*_0_ a constant that depend on the exact choice of the mother wavelet *ψ*_0_ and the sampling frequency *f*_*s*_ (in the present work, given that orthonormal Daubechies wavelets are used *f*_0_ ≃ 3 ⋅ *f*_*s*_/4) blackbecause of their excellent localization in the frequency domain. This thus justifies the practical approximation used in Eq (4).

Plugging a power-law-shaped PSD, as in Eq (1), into Eq (11) directly yields the power law behavior of the wavelet spectrum as in Eq (3). Further, the definition of the LF/HF ratio reads:

$$\begin{array}{c} LF/HF_{f_{\min},f_{interm},f_{\max}}=\frac{\int_{f_{\min}}^{f_{interm}}\Gamma_{X}\left(f\right)a{\left|\Psi_{0}\left(af\right)\right|}^{2}df}{\int_{f_{interm}}^{f_{\max}}\Gamma_{X}\left(f\right)a{\left|\Psi_{0}\left(af\right)\right|}^{2}df}. \end{array}$$ (12)

Plugging in a power-law decaying PSD, as modeled in Eq (1), yields a formal connection between *H* and the LF/HF ratio:

$$\begin{array}{c} LF/HF_{f_{\min},f_{interm},f_{\max}}=\frac{\int_{f_{\min}}^{f_{interm}}{C|f|}^{-(2H-1)}a{\left|\Psi_{0}\left(af\right)\right|}^{2}df}{\int_{f_{interm}}^{f_{\max}}{C|f|}^{-(2H-1)}a{\left|\Psi_{0}\left(af\right)\right|}^{2}df}=\frac{f_{interm}^{2-2H}-f_{\min}^{2-2H}}{f_{\max}^{2-2H}-f_{interm}^{2-2H}}. \end{array}$$ (13)

## Supporting Information

S1 TableS1 Table provides, for each case, the following information: Case ID, Group (Control, Index), CTG evaluation (Normal, Abnormal), basal heart rate (bpm), long-term variability (bpm), short-term variability (ms), absolute and normalized powers for LF and HF bands and LF/HF ratio, representative LF/HF_*f*_*interm*__ ratios computed from ranges LF = [0.04, *f*_*interm*_], HF = [*f*_*interm*_, 0.40] with different intermediate frequencies *f*_*interm*_ ∈ {0.1, 0.15, 0.2, 0.25, 0.30, 0.35}, and the Hurst parameter estimated at scales corresponding to the particular frequency range ${\hat{H}}_{0.04-0.40}$ and ${\hat{H}}_{0.02-1.25}$.(ZIP)
